# Identification of Biomarker in Brain-specific Gene Regulatory Network Using Structural Controllability Analysis

**DOI:** 10.3389/fbinf.2022.812314

**Published:** 2022-01-26

**Authors:** Zhihua Chen, Siyuan Chen, Xiaoli Qiang

**Affiliations:** ^1^ The Institute of Computing Science and Technology, Guangzhou University, Guangzhou, China; ^2^ The School of Artificial Intelligence and Automation, Huazhong University of Science and Technology, Wuhan, China

**Keywords:** brain-specific gene regulatory network, controllability analysis, biomarker, brain tumor research, robustness analysis

## Abstract

Brain tumor research has been stapled for human health while brain network research is crucial for us to understand brain activity. Here the structural controllability theory is applied to study three human brain-specific gene regulatory networks, including forebrain gene regulatory network, hindbrain gene regulatory network and neuron associated cells cancer related gene regulatory network, whose nodes are neural genes and the edges represent the gene expression regulation among the genes. The nodes are classified into two classes: critical nodes and ordinary nodes, based on the change of the number of driver nodes upon its removal. Eight topological properties (out-degree *DO*, in-degree *DI*, degree *D*, betweenness *B*, closeness *CA*, in-closeness *CI*, out-closeness *CO* and clustering coefficient *CC*) are calculated in this paper and the results prove that the critical genes have higher score of topological properties than the ordinary genes. Then two bioinformatic analysis are used to explore the biologic significance of the critical genes. On the one hand, the enrichment scores in several kinds of gene databases are calculated and reveal that the critical nodes are richer in essential genes, cancer genes and the neuron related disease genes than the ordinary nodes, which indicates that the critical nodes may be the biomarker in brain-specific gene regulatory network. On the other hand, GO analysis and KEGG pathway analysis are applied on them and the results show that the critical genes mainly take part in 14 KEGG pathways that are transcriptional misregulation in cancer, pathways in cancer and so on, which indicates that the critical genes are related to the brain tumor. Finally, by deleting the edges or routines in the network, the robustness analysis of node classification is realized, and the robustness of node classification is proved. The comparison of neuron associated cells cancer related GRN (Gene Regulatory Network) and normal brain-specific GRNs (including forebrain and hindbrain GRN) shows that the neuron-related cell cancer-related gene regulatory network is more robust than other types.

## 1 Introduction

The world has opened its eyes to the threat posed by cancer (McGuire, 2016). Brain tumor is a mass or growth of abnormal cells in the human brain. It can begin in the human brain (primary brain tumors), or begin in other parts of body and spread to brain (secondary, or metastatic, brain tumors) (Cheng et al. 2014). Brain tumor accounts for 85–90% of all primary central nervous system (CNS) tumors (Mehta et al., 2011). There are new cases and deaths from brain tumor and other nervous system tumors estimated around the world every year. Approximately 256,213 new cases of brain and other CNS tumors were diagnosed in the year 2012, with an estimated 189,382 deaths (Ferlay et al., 2012), and there are 296,851 new cases and 241,037 deaths in 2018 (Bray et al., 2018). Furthermore, the cause of most adult brain and spinal cord tumors is not known. It is urgent to study the pathogenic mechanism and treatment for brain tumors.

Many studies have focused on the role of single molecule or single pathway in regulating tissue-specific nuclear structure and gene expression. For example, SATB1, a cell type specific nuclear protein, can recruit chromatin remodeling factors and regulate many genes during thymocyte differentiation. And it is proposed by Cai-s et al. as a novel gene regulator, which can provide sites for tissue-specific and region specific histone modification of DNA sequences (Cai et al., 2003). Fass et al. (2018) studied the role of GPCR-kinase interacting protein 1 (GIT1), and found that GIT1 deletion interferes with the specific network of GIT1 interacting synapses. Although the understanding of individual molecules is crucial, the focus is on understanding the entire gene regulatory network at the system level. Because the properties of gene regulatory networks cannot be fully understood by studying single molecules (Kitano, 2002).

Marbach D et al. Developed a comprehensive resource of 394 cell types and tissue-specific gene regulatory networks with 37 genome wide association studies (GWAS), which clarifies the genome-wide connectivity among transcription factors, enhancers, promoters and genes (Marbach et al., 2016). McKenzie A T et al. identified a novel set of brain cell consistent signatures and robust networks from the integration of multiple data sets, so it goes beyond the limitations associated with each individual research specific technical problem (McKenzie et al., 2018). Therefore, it is feasible to construct brain specific gene regulatory network based on relevant data and it is effective to study it based on network analysis.

Network Science has become an emerging and highly interdisciplinary research area that aims to increase our understanding of complex networks (Barabási, 2009; Börner et al., 2007; Liu et al., 2016; Gao et al., 2015; Peng et al., 2015). At the same time, with the increasing of massive genomic, proteomic, and metabolomics data, the formation of multi-layer biological molecular network is promoted, which lays a foundation for the analysis of biological problems by network science (Liu et al., 2019b; Ortmayr et al., 2019; Malod-Dognin et al., 2019; Liu et al., 2019a). Detailed maps of mammalian brains could lead to a revolution in brain science, which allows us to understand and find the cure of numerous neurological and brain diseases. With that, network science could be applied in brain research widely (Sporns et al., 2005; Barabási and Pósfai, 2016; Liu and Pan, 2016b). It has been applied in many kinds of biological networks, such as mouse inter-region brain networks and human transcription factor regulatory networks (Chang, 2015). Liu et al. used the control theory of structural controllability to analyse numerous models of real networks, for instance, the directed human protein interaction network, which helped us to identify disease genes and drug targets (Vinayagam et al., 2016), the human signaling network to identify driver nodes (Liu and Pan, 2015) and so on. It has been proved that a system’s behaviour can be guided towards a desired state with a suitable choice of control signals to some significant nodes (Liu and Pan, 2016a; Liu et al., 2011; Yan et al., 2017; Liu et al., 2017). Therefore, it’s feasible that we can apply controllability theory of network science on the analysis of brain-specific gene regulatory network.

Network structural controllability analysis (Liu et al., 2011, Liu et al., 2017) has been a general framework in identifying critical nodes that have crucial roles in controlling the state of the whole system. By applying this framework to human liver metabolic networks (Liu and Pan, 2014), the critical driver metabolites tend to be essential. Moreover, this framework can be used to predict potential drug-targets (Liu and Pan, 2015). It is fair to expect that there are some possible connections between the structural controllability theory and the human brain-specific gene regulatory networks, which could provide valuable informations on the brain-specific gene regulatory networks, such as identifying essential genes, brain-related disease genes and drug targets.

In this work, we apply structural controllability method to analyse large-scale directed human brain-specific gene regulatory networks, where nodes are neurons and edges represent the gene expression regulation among the genes. The weight of each edge ranges from 0 to 1, which measures the normalized activity levels of the enhancer. We classify the nodes into two classes: critical genes and ordinary genes. Then we do the topological and biological analyse of these two kinds of genes, and find that critical genes tend to be essential genes. By calculating eight topological properties (out-degree, in-degree, degree, betweenness, closeness, in-closeness, out-closeness and clustering coefficient), we can see that critical genes have higher score of topological properties than ordinary genes. Moreover, the enrichment in several kinds of gene databases is explored, which shows that critical nodes are richer in essential gene, cancer gene and the neuron related disease gene than ordinary nodes. Besides, GO analysis and KEGG pathway analysis also help to infer that critical nodes are useful for us to explore more significant biological information and to identify the biomarker for brain tumor research. Finally, since the gene regulatory network is not complete or there may be some false links, we do sensitivity analysis of the results by perturbing the network. We find that the result of node classification is quite robust, and the neuron associated cells cancer related gene regulatory network is more robust than the health networks. The findings in our paper could help identify potential essential, cancer and neuron related disease genes.

## 2 Results

### 2.1 Classification by Driver Nodes

#### 2.1.1 Description of Brain-specific Gene Regulatory Network

We construct three brain-specific gene regulatory networks, which consist of forebrain gene regulatory network (forebrain GRN), hindbrain gene regulatory network (hindbrain GRN) and neuron associated cells cancer related gene regulatory network (neuron associated cells cancer GRN). They are directed networks and their nodes are neuronal genes. Their edges represent the regulation among neuronal genes, specifying the genome-wide connectivity among transcription factors, enhancers, promoters and genes (Marbach et al., 2016). Forebrain GRN and hindbrain GRN are normal and healthy human’s brain-specific gene regulatory networks, while neuron associated cells cancer related GRN is cancer patient’s brain-specific gene regulatory network. In these networks, the edge direction corresponds to the hierarchy of signal flow between the interacting genes and the edge weight corresponds to the confidence of the predicted direction. Generally, we delete the edges whose edge weight is smaller than 0.05 for our study. By this way, the human forebrain GRN consists of 14,435 genes (nodes) and 2,22,867 directed edges, the human hindbrain GRN consists of 14,601 genes and 2,28,708 directed edges, and the neuron associated cells cancer related GRN consists of 15,320 genes and 2,56,434 edges.

#### 2.1.2 Classification of Brain-specific Gene Regulatory Networks’ Nodes

By structural controllability theory, the minimum set of driver nodes is identified and the size of it is calculated as *N*_*D*_. Then the nodes are classified as critical or ordinary, based on the change of *N*_*D*_ upon their removal. The node is critical if *N*_*D*_ has no change or increases because of its removal, or the node is ordinary if *N*_*D*_ decreases. The results are shown in Table 1. The number of forebrain GRN’s critical nodes is 642, while the number of hindbrain GRN’s and neuron associated cells cancer related GRN’s critical nodes are both 643.

**TABLE 1:** Classification by driver nodes.

Network	Critical	Number of nodes	Total
		Ordinary	
Forebrain GRN	642*(* ***4.45%*** *)*	13,793	14,435
Hindbrain GRN	643*(* ***4.40%*** *)*	13,958	14,601
Neuron associated cells cancer GRN	643*(* ***4.20%*** *)*	14,677	15,320

### 2.2 Topological Analysis

Different centralities of each gene in the three brain-specific gene regulatory networks are calculated, which including out-degree *DO*, in-degree *DI*, degree *D*, betweenness *B*, closeness *CA*, in-closeness *CI*, out-closeness *CO*, betweenness *B* and clustering coefficient *CC*. The average values of each topological property are shown in Table 2. It is clear that there are some similar topological properties among the three brain-specific gene regulatory networks, so we can make topological analysis from two aspects.

**TABLE 2:** Topological analysis of each gene in human brain network.

Forebrain GRN		Ordinary	Hindbrain GRN	Ordinary	Neuron associated	cells cancer GRN
Critical			Critical		Critical	Ordinary
D	364.402	15.354	375.426	15.476	425.121	16.319
DI	17.257	15.355	19.737	15.476	26.313	16.318
DO	347.145	0	355.689	0	398.809	0
B	25,212.564	0	25,862.054	0	26,124.840	0
CA	2.912e-05	2.779e-05	2.924e-05	2.758e-05	2.872e-05	2.661e-05
CI	4.943e-09	5.008e-09	4.848e-09	4.898e-09	4.407e-09	4.439e-09
CO	1.987e-07	4.800e-09	2.449e-07	4.690e-09	3.012e-07	4.260e-09
CC	0.029	0.279	0.034	0.275	0.040	0.297

**FIGURE 1:**
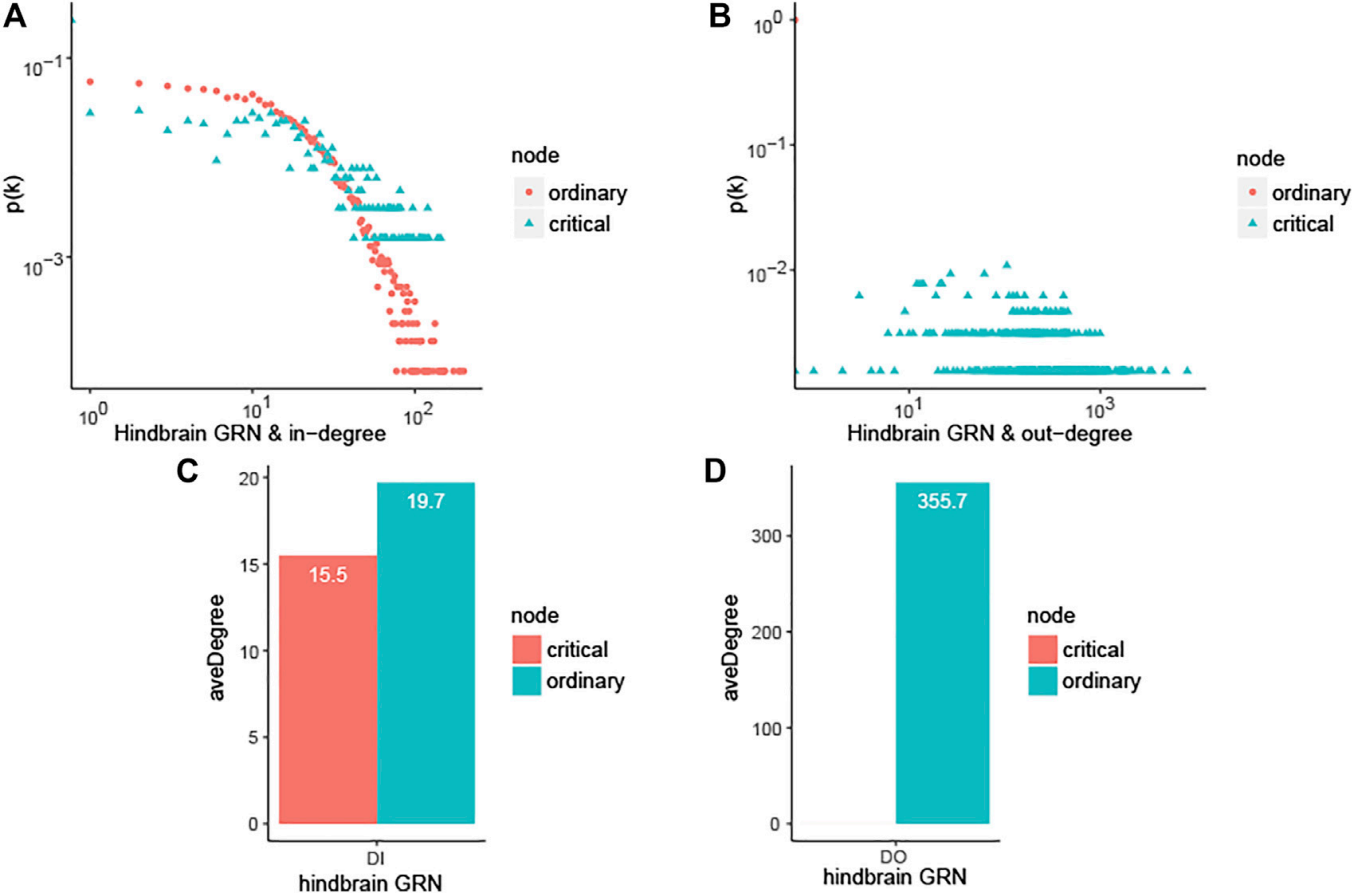
Characterizing the controllability of human brain-specific gene regulatory network and topological analysis. In the figure, hindbrain GRN is hindbrain gene regulatory network, forebrain GRN is forebrain gene regulatory network, neuron associated cells cancer related GRN is neural gene regulatory network associated cells cancer (Table 2).The values in Table 2 are the average of those topological characteristics that consist of out-degree *DO*, in-degree*DI*, degree *D*, betweenness *B*, closeness *CA*, in-closeness *CI*, out-closeness *CO*, betweenness *B* and clustering coefficient *CC*. **(A)** In-degree distribution of hindbrain GRN. **(B)** Out-degree distribution of hindbrain GRN. **(C)** Average in-degree for ordinary and critical nodes in hindbrain GRN. **(D)** Average out-degree for ordinary and critical nodes in hindbrain GRN.

On one aspect of single network, taking the hindbrain GRN into account, the ordinary nodes’ out-degree is 0, as shown in Figures 1B,D, which means that the ordinary genes in the networks are all just signal receivers. And the degree distribution suggests that critical nodes have higher degree than ordinary nodes as shown in Figure 1, (degree distribution of forebrain GRN and neuron associated cells cancer related GRN are shown in Supplementary Figure S1, and it shows the same result as hindbrain GRN). The average of the ordinary nodes’ betweenness is also 0. What’s more, the average out-closeness of critical nodes is much bigger than ordinary nodes, and it is proved convincing by significance testing (Mann Whitney U test, *p*-value is smaller than 0.05, significance level = 0.05, see Table 3). These results imply that critical nodes are more important or useful for further study.

**TABLE 3:** The significance test of *CO*.

Network	Mann Whitney U test	W
	*p*-value	
Forebrain GRN	2.2e-16	86,96,500
Hindbrain GRN	2.2e-16	88,70,300
Neuron associated cells cancer GRN	2.2e-16	93,56,600

On the other aspect of comparison of the three brain-specific gene regulatory networks, the average in-degree, average out-degree, average betweenness and average clustering coefficient of neuron associated cells cancer related GRN are bigger than forebrain GRN and hindbrain GRN, no matter critical or ordinary nodes. It can be explained that the connection of neuron associated cells cancer related GRN is tighter than the normal brain network.

### 2.3 Biological Enrichment Analysis

The enrichment scores of the critical genes and ordinary genes in different biofunctional gene databases are calculated, and GO analysis and KEGG pathway analysis are adopted on critical genes to explore the biological significance.

#### 2.3.1 Enrichment Score Calculating Analysis

**FIGURE 2:**
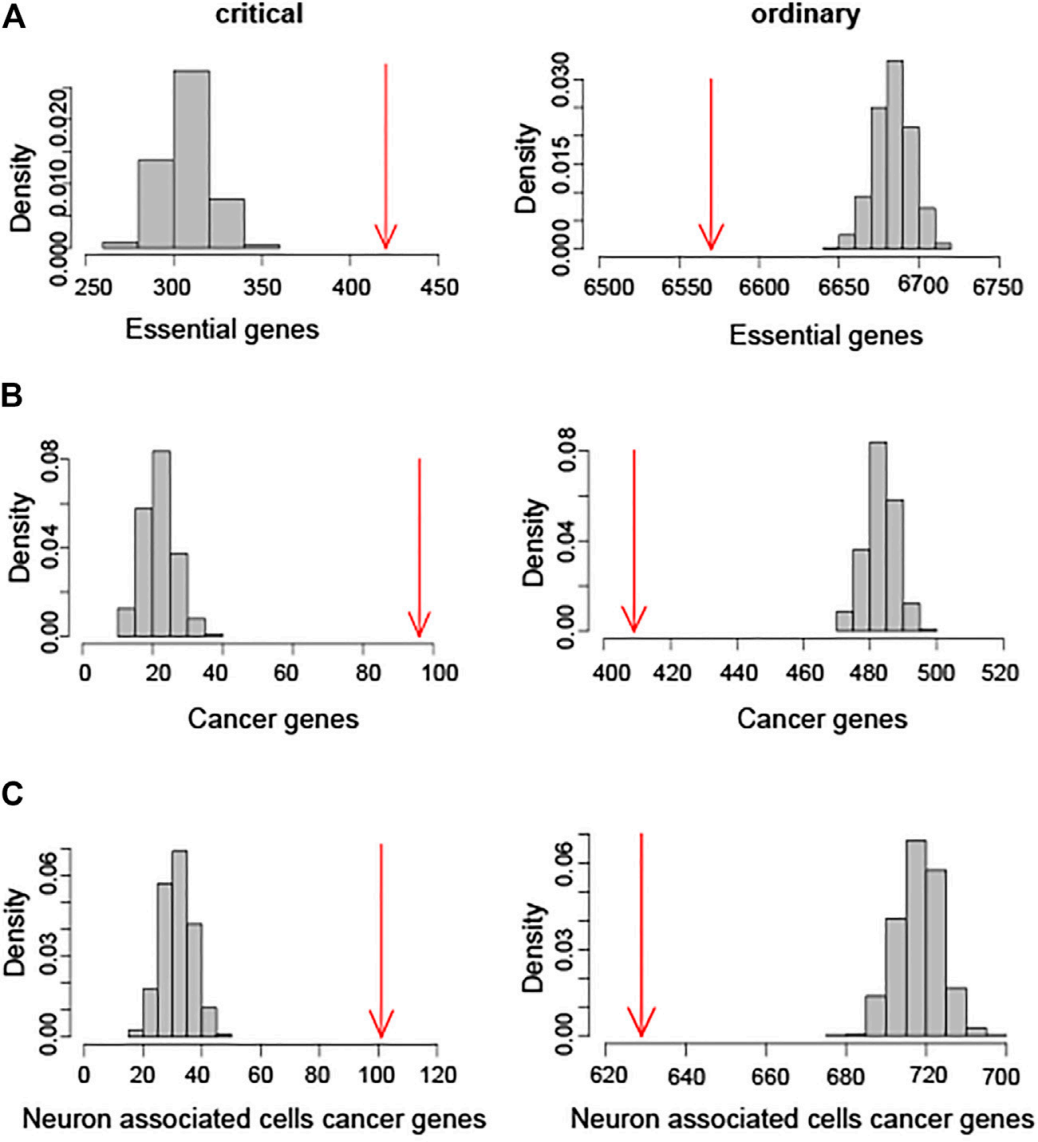
Biological enrichment score calculating analysis of hindbrain GRN. **(A)** Enrichment analysis of essential genes. Numbers of essential genes overlapping with critical and ordinary nodes are shown in red arrows. **(B)** Enrichment analysis of cancer genes. Numbers of cancer genes overlapping with critical and ordinary nodes are shown in red arrows. **(C)** Biological enrichment analysis of neuron associated cells cancer genes. Numbers of these genes overlapping with critical and ordinary nodes are shown in red arrows.

The nodes in the databases which consist of essential genes, cancer genes, and the neuron related disease genes respectively are characterized as critical and ordinary nodes. Essential genes are necessary for cellular survivor. The gene essentiality analysis indicates that critical nodes are enriched in essential genes, whereas essential genes are underrepresented among ordinary nodes (Figure 2). Furthermore, the critical nodes are enriched in cancer genes and the related disease genes in the Figure 2, and it indicates that the disease genes are most likely among the critical nodes. Supplementary Figures S2, S3 show the biological enrichment analysis of forebrain GRN and neuron associated cells cancer related GRN, respectively. These results indicate that the proposed classification method is a reliable and useful tool for the prediction of brain tumor biomarkers. The critical genes mined in this way have bioinformatics significance and have a strong correlation with brain tumors.

#### 2.3.2 GO Analysis and KEGG Pathway Analysis on Critical Genes

GO (Gene Ontology) analysis (du Plessis et al., 2011) has the largest resource for cataloguing gene function, which is subdivided into three non-overlapping ontologies, Molecular Function (MF), Biological Process (BP) and Cellular Component (CC). KEGG (Kyoto Encyclopedia of Genes and Genomes) (Kanehisa and Goto, 2000) is a knowledge base for systematic analysis of gene functions, linking genomic information with higher order functional information. More detailed biological function of the genes can be obtained from GO analysis and KEGG pathway analysis.

**FIGURE 3:**
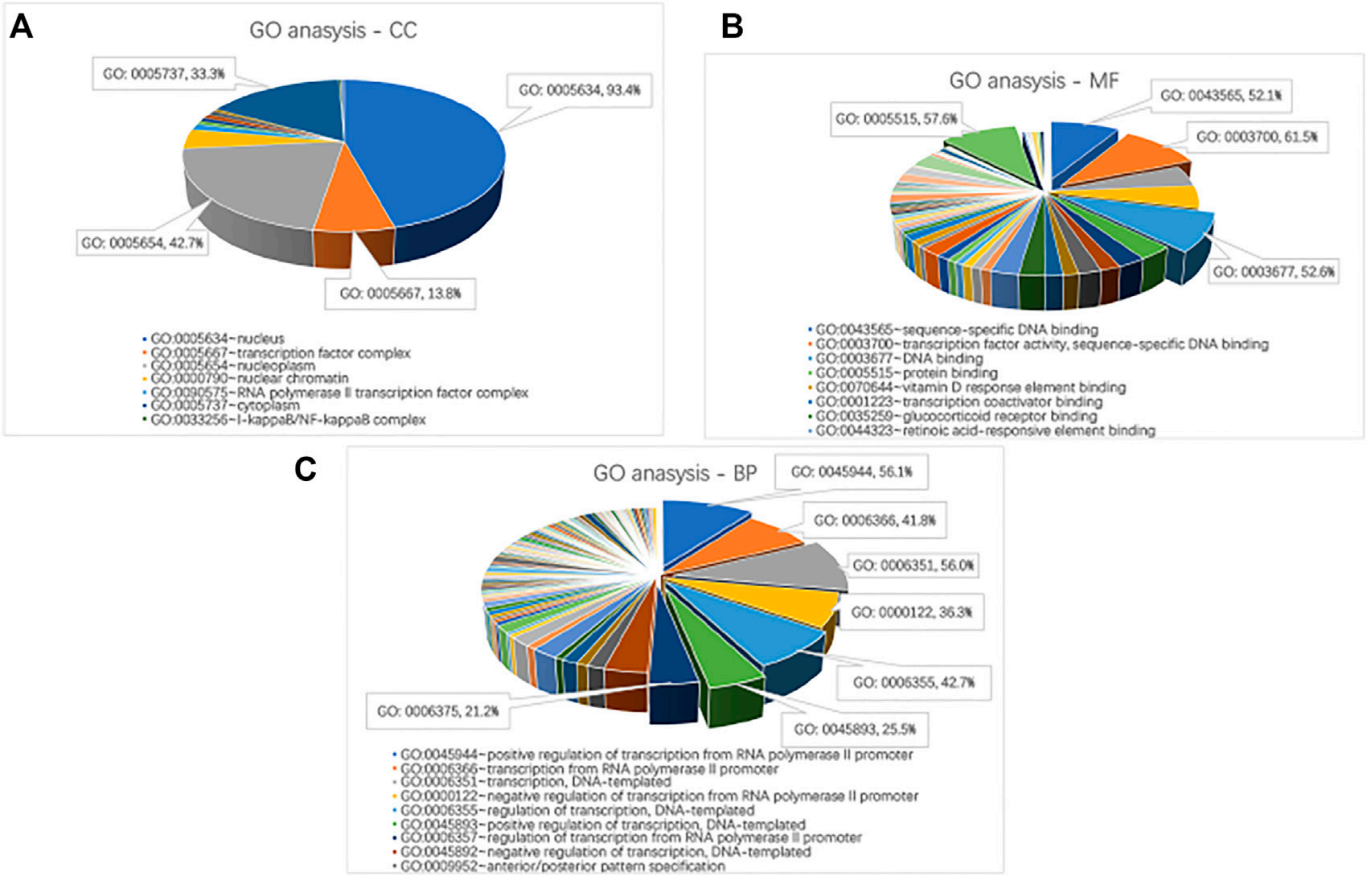
Go analysis on critical genes. **(A)** CC. **(B)** MF. **(C)** BP.

David, the online biological enrichment analysis software, is used to realize the GO analysis and KEGG pathway analysis on critical genes. The results are shown in Figure 3 and Table 4. GO analysis points out that critical genes are rich in nucleus (93.4%), nucleoplasm (42.7%), cytoplasm (33.3%) and so on. Their molecular functions mostly consist of transcription factor activity, sequence-specific DNA binding and protein binding. What’s more, the critical genes mainly play important roles in 7 biological processes, which are positive regulation of transcription from RNA polymerase II promoter (56.1%), DNA-templated (56.0%), etc. KEGG pathway analysis shows that the critical genes are rich in 14 KEGG pathways, which are confirmed with brain tumor from the Comparative Toxicogenomics Database (CTD, http://ctdbase.org/).

**TABLE 4:** KEGG Pathway analysis on critical genes.

KEGG Pathway	Gene Number	*p* value	Genes
hsa05202:Transcriptional misregulation in cancer	45*(* ***7.56%*** *)*	1.58e-32	LMO2, TFE3, PPARG,…
hsa05166:HTLV-I infection	33*(* ***5.55%*** *)*	1.32e-13	STAT5A, SPI1, ELK1,…
hsa05200:Pathways in cancer	40*(* ***6.72%*** *)*	5.29e-13	PPARG, SPI1, FOXO1,…
hsa04550:Signaling pathways regulating			
pluripotency of stem cells	24*(* ***4.03%*** *)*	1.89e-12	NANOG, HNF1A, OTX1,…
hsa05203:Viral carcinogenesis	24*(* ***4.03%*** *)*	4.99e-09	EGR3, EGR2,SP100,…
hsa05030:Cocaine addiction	9*(* ***1.51%*** *)*	4.29e-05	ATF4, CREB3, RELA,…
hsa04390:Hippo signaling pathway	15*(* ***2.52%*** *)*	5.27e-05	TCF7, SOX2, SMAD4,…
hsa04919:Thyroid hormone signaling pathway	13*(* ***2.18%*** *)*	5.80e-05	THRA, THRB, RXRB,…
hsa04022:cGMP-PKG signaling pathway	14*(* ***2.35%*** *)*	3.25e-04	MEF2D, MEF2B, ATF4,…
hsa04668:TNF signaling pathway	10*(* ***1.68%*** *)*	0.002 33	ATF4, CEBPB, CREB3,…
hsa04152:AMPK signaling pathway	10*(* ***1.68%*** *)*	0.005 92	SREBF1, HNF4A, CREB3,…
hsa04068:FoxO signaling pathway	10*(* ***1.68%*** *)*	0.010 20	EP300, FOXG1, SMAD4,…
hsa04310:Wnt signaling pathway	10*(* ***1.68%*** *)*	0.012 22	TCF7, EP300, TP53,…
hsa04110:Cell cycle	9*(* ***1.51%*** *)*	0.018 82	E2F4, EP300, TP53,…

From the above analysis, we can find that the critical genes we find are important and have an association with brain tumor. And GO analysis and KEGG pathway analysis provide the reference for further study on brain tumor.

### 2.4 Robustness Analysis of Node Classification

Since the critical nodes are vital and significant, it’s necessary to know whether the classification is robustness if the network is attacked. Therefore, the robustness of node classification is systematically tested by deleting edges or nodes.

#### 2.4.1 Deleting Edges

**FIGURE 4:**
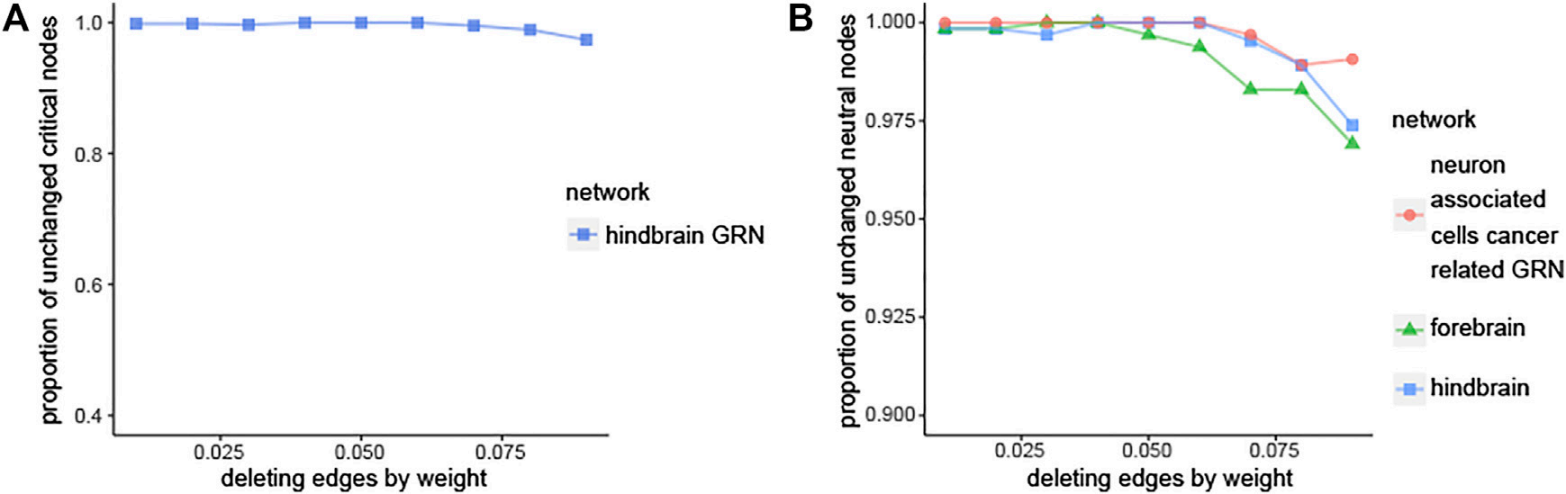
Robustness analysis of node classification. **(A)** Plot showing the fraction of ciritical nodes in new networks that overlaps with the original hindbrain GRN, the new networks are obtained by deleting edges from original hindbrain gene regulatory networks. **(B)** The comparison of three networks in case of deleting edges.

Over 96% edges’ weights are between 0 and 0.10 in the three brain-specific gene regulatory networks. The detailed information can be seen in Table 5. Therefore, the edges, whose edge weight are smaller than 0.01, 0.02, … , 0.10, are deleted respectively to get new networks. Then the same method is used to identify the new networks’ critical nodes and ordinary nodes. Finally, the proportions of the critical nodes of the new networks to the original network are compared in Figure 4A and Supplementary Figure S4. It can be seen that the node classification is robust with respect to deleting edges in the three brain-specific gene regulatory networks. What’s more, through comparing the three curves in Figure 4B, we can know that the neuron associated cells cancer related GRN has higher value than the others, which means the neuron associated cells cancer related GRN is more robust than the health brain-specific gene regulatory networks.

**TABLE 5:** The Edge weight ratios of three GRN networks.

Network	Number of edges	edge weight (0 ∼0.10)
	All	
Forebrain GRN	26,62,324	25,91,577*(* ***97.3%*** *)*
Hindbrain GRN	26,06,819	25,33,623*(* ***97.2%*** *)*
Neuron associated cells cancer GRN	26,54,287	25,65,486*(* ***96.7%*** *)*

#### 2.4.2 Deleting Nodes by Critical Nodes

**FIGURE 5:**
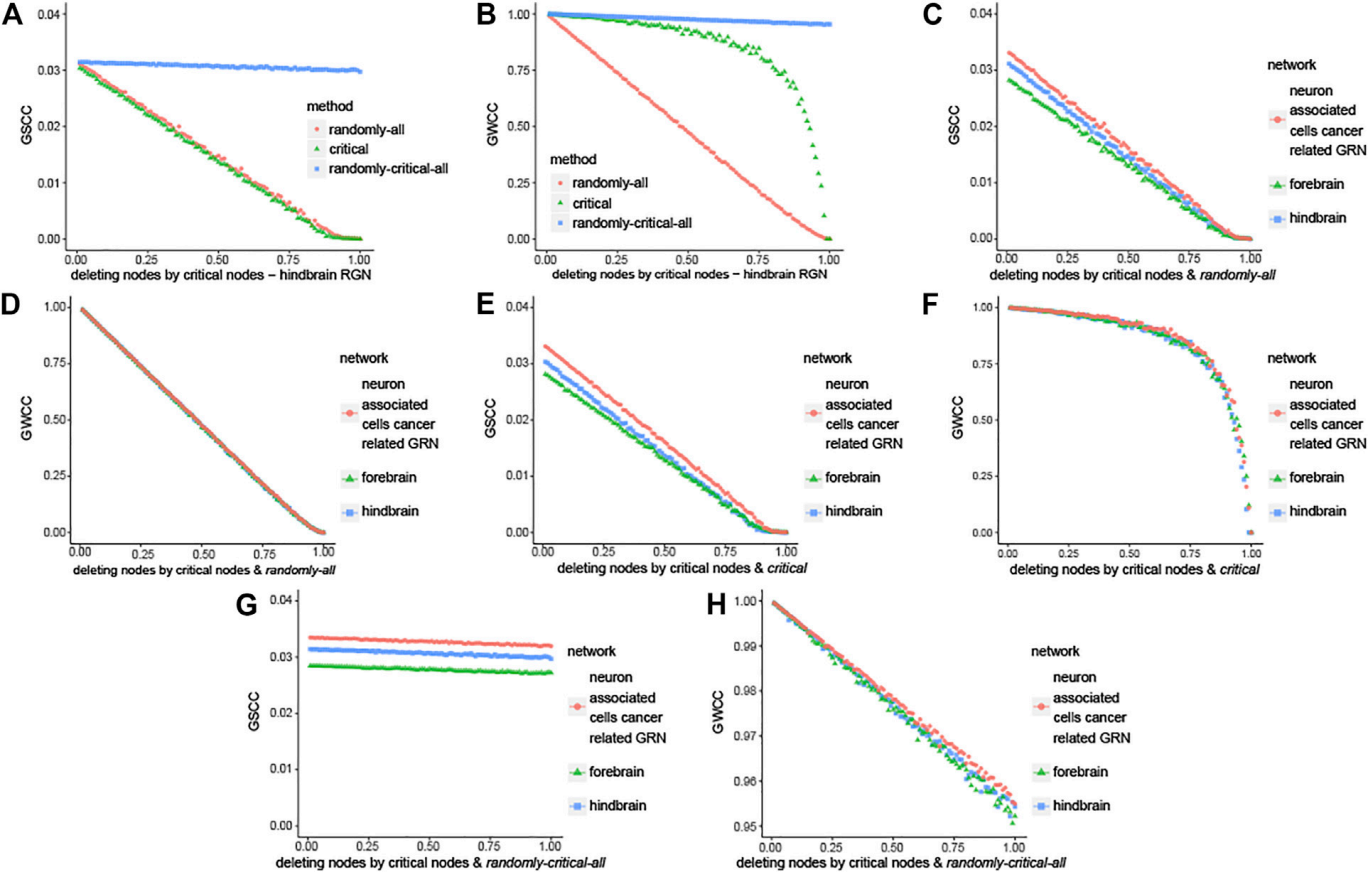
Robustness analysis of node classification. Here we noted the three strategies by the following ways: Firstly, deleting nodes randomly in the whole hindbrain GRN (noted as *randomly—all*). Secondly, deleting critical nodes randomly (noted as *critical*). Lastly deleting the same number of nodes as the second strategy in the whole hindbrain GRN, and which nodes are deleted is randomly (noted as *randomly—critical—all*). **(A)** The change of giant strongly connected component (GSCC) in hindbrain GRN with three methods that are different in the way of deleting critical nodes. **(B)** The change of giant weakly connected component (GWCC) in hindbrain GRN with three methods that are different in the way of deleting critical nodes. **(C, D)** Comparison in the way of deleting nodes randomly in the whole brain-specific GRN respectively. **(E, F)** Comparison in the way of *critical*. **(G, H)** Comparison in the way of *randomly—critical—all*.

In this part, the change of giant strongly connected component (GSCC) and giant weakly connected component (GWCC) are compared under three deleting strategies. It is applied to the three brain-specific gene regulatory networks in the same way, so we take hindbrain GRN for example. Firstly, deleting nodes randomly in the whole hindbrain GRN (noted as *randomly—all*). Secondly, deleting critical nodes randomly (noted as *critical*). Finally, deleting the same number of nodes as the second strategy in the whole hindbrain GRN, and whose nodes are deleted is randomly (noted as *randomly—critical - all*). Compared the curve of *critical* with *randomly—all* and *randomly—critical—all*, the results are shown in Figures 5A,B indicate that the maximal connected subgraph becomes smaller and smaller with the decrease of the critical nodes. It means that the critical nodes are crucial for the connection of the whole network. It is consistent with the results of forebrain GRN and neuron associated cells cancer related GRN from Supplementary Figure S5. Moreover, the maximal connected subgraph is bigger than the others as described in Figures 5C–H, which are the comparisons among three brain networks in the same strategy. It means that neuron associated cells cancer related GRN is more robust than normal and healthy brain-specific gene regulatory network.

## 3 Discussion

In this paper, the genes in human brain-specific genes regulatory networks (forebrain GRN, hindbrain GRN and neuron associated cells cancer related GRN) are divided into the critical genes and ordinary genes. By calculating eight topological properties (out-degree *DO*, in-degree *DI*, degree *D*, betweenness *B*, closeness *CA*, in-closeness *CI*, out-closeness *CO* and clustering coefficient *CC*), we find that the critical genes play important roles in the human brain-specific GRN networks. For example, the critical genes have larger score of *CO* than the ordinary genes. Biological enrichment analysis in essential genes database shows that critical genes are richer than the ordinary genes, so we predict that critical genes are more significant for us to explore biological information. Furthermore, the enrichments in cancer genes database and neuron related disease genes database are explored, and it is consistent with our prediction. Because the critical nodes are richer than the ordinary nodes in these gene databases. It indicates that the critical genes can contribute to identifying the disease genes related to brain. GO analysis and KEGG pathway analysis indicate that critical genes are associated with brain tumor and hint that they are rich in transcriptional misregulation in cancer, pathways in cancer and so on, which provide references for further study on brain tumor. Finally, tests show that the nodes classification method is robust when the network is attacked. And the tests indicate that the neuron associated cells cancer related GRN is more robust than normal and healthy brain-specific gene regulatory networks (forebrain GRN and hindbrain GRN), which is straightaway that a person gets sick easily but regains health difficultly.

In conclusion, controllability theory is also a useful tool to analyse human brain-specific gene regulatory network. It can provide a feasible direction for biologists to study whether the biomarker mined by the proposed method is related to brain tumor or not. In addition, the research work also raises a number of questions. For instance, how can we quantify the influence of each critical genes for the network? Can the work expand to the structure of function brain network? Answers to these questions can further provide theoretical foundation for designing experiments.

## 4 Methods

### 4.1 Brain-specific Gene Regulatory Networks

There are three kinds of brain-specific gene regulatory networks. For convenience, we called them hindbrain GRN, forebrain GRN and neuron associated cells cancer related GRN respectively. Hindbrain GRN and forebrain GRN are normal and healthy adult’s brain-specific gene regulatory networks, while the neuron associated cells cancer related GRN is the patient’s brain-specific gene regulatory network, who suffers from brain tumor. They all are dealt with by deleting edges whose edge weights are smaller than 0.05 before we use them to analyse in this paper.

### 4.2 Structural Controllability and Its Applications to Biological Networks

Biological networks are complex nonlinear systems. The controllability of nonlinear systems is structurally similar to that of linear systems (Slotine and Li, 1991; Liu et al., 2011). We study a system with canonical linear, time-invariant dynamics formulated by Lombardi and Hörnquist (2007).

$$\frac{dx\left(t\right)}{dt}=Ax\left(t\right)+Bu\left(t\right),$$ (1)

where the vector $x\left(t\right)={\left(x_{1}\left(t\right),x_{2}\left(t\right),\ldots ,x_{N}\left(t\right)\right)}^{T}$ describes the states of the *N* nodes of the networked system at time *t*. The *N* × *N* matrix *A* is the transposition of the adjacency matrix and captures the wiring diagram of the system and the interaction strengths between nodes. The *N* × *M* matrix *B* is the input matrix (*N* ≥ *M*) that identifies the nodes into which the input signals are injected, *M* is the number of input signals, and $u\left(t\right)={\left(u_{1}\left(t\right),u_{2}\left(t\right),\ldots ,u_{\text{m}}\left(t\right)\right)}^{T}$ is the input vector.

In control theory, a system is controllable if it can be driven from any initial state to any desired final state during a finite time period (Kalman, 1963). According to Kalman’s controllability rank condition (Kalman, 1963), the system represented by Eq. 1 is controllable if and only if the *N* × *NM* controllability matrix *C* has full rank, i.e.,

$$rank\left(C\right)=rank\left[B,AB,A^{2}B,\ldots ,A^{N-1}B\right]=N.$$ (2)

This controllability rank condition indicates that to control the full network we must identify the number of signals and the nodes into which the signals are injected, called driver nodes. Liu et al. (2011) recently showed that a full system can be structurally controlled by inputting signals into a minimum set of driver nodes. A system is structurally controllable if it is possible to choose non-zero weights in *A* and *B* such that Eq. 2 holds (Liu et al., 2011). The minimum number of driver nodes for controlling a full network is denoted *N*_*D*_ and the minimum driver node density is *n*_*D*_ = *N*_*D*_/*N*. The minimum driver node density required to control the full complex network quantifies its structural controllability (Liu et al., 2011; Liu et al., 2015).

Structural controllability analysis has been applied to some biological networks, where interesting properties on the biological system and drug-targets have been discovered (Liu and Pan, 2014; Liu and Pan, 2015; Vinayagam et al., 2016). According to the frequency of appearing in the minimum driver node sets, or the impact of removing a node on the minimum number of driver nodes, the nodes can be classified in to different classes: critical, redundant and ordinary (as explained in the following subsection). In biological molecular networks, biological molecules can be classified with different roles. By doing biological enrichment analysis of these different biological roles, candidate essential genes or drug-targets can be identified.

### 4.3 Node Classification

According to the control theory, a dynamical system is controllable if, with a suitable choice of inputs, it can be driven from any initial state to any desired final state within finite time (Kalman, 1963; Luenberger, 1979). By using the analytical tool developed by Liu et al. (2011), we can identify the set of driver nodes in an arbitrary complex directed network, with time-dependent control that can guide the systems entire dynamics to study its controllability. Moreover, the minimum number of driver nodes is determined for a determined network. Hence, the mathematical framework and analytical tools that have been developed by Vinayagam et al. (2016) can be used to compute the minimum number, and denote it as *N*_*D*_. After removing a node, we denote the minimum number of driver nodes of the damaged network as $N_{D}^{\prime }$ . Then we classify the node by comparing *N*_*D*_ and $N_{D}^{\prime }$ . A node is critical if $N_{D}^{\prime }>N_{D}$ or $N_{D}^{\prime }=N_{D}$ , and ordinary if $N_{D}^{\prime }<N_{D}$ . For example, hindbrain GRN’s *N*_*D*_ = 13 959, the deleted node is critical if $N_{D}^{\prime }=13\;959$ , ordinary if $N_{D}^{\prime }=13\;958$ .

### 4.4 Biological Enrichment Score Calculating Analysis

Biological enrichment score calculating analysis is a method for enrichment analysis of gene sets, which is used to identify gene classes that are over-expressed in a large group of genes and may be related to disease phenotypes. This method uses statistical methods to identify significantly enriched or missing genomes. Microarray and proteomic results usually identify thousands of genes for analysis (Subramanian et al., 2005).

**FIGURE 6:**
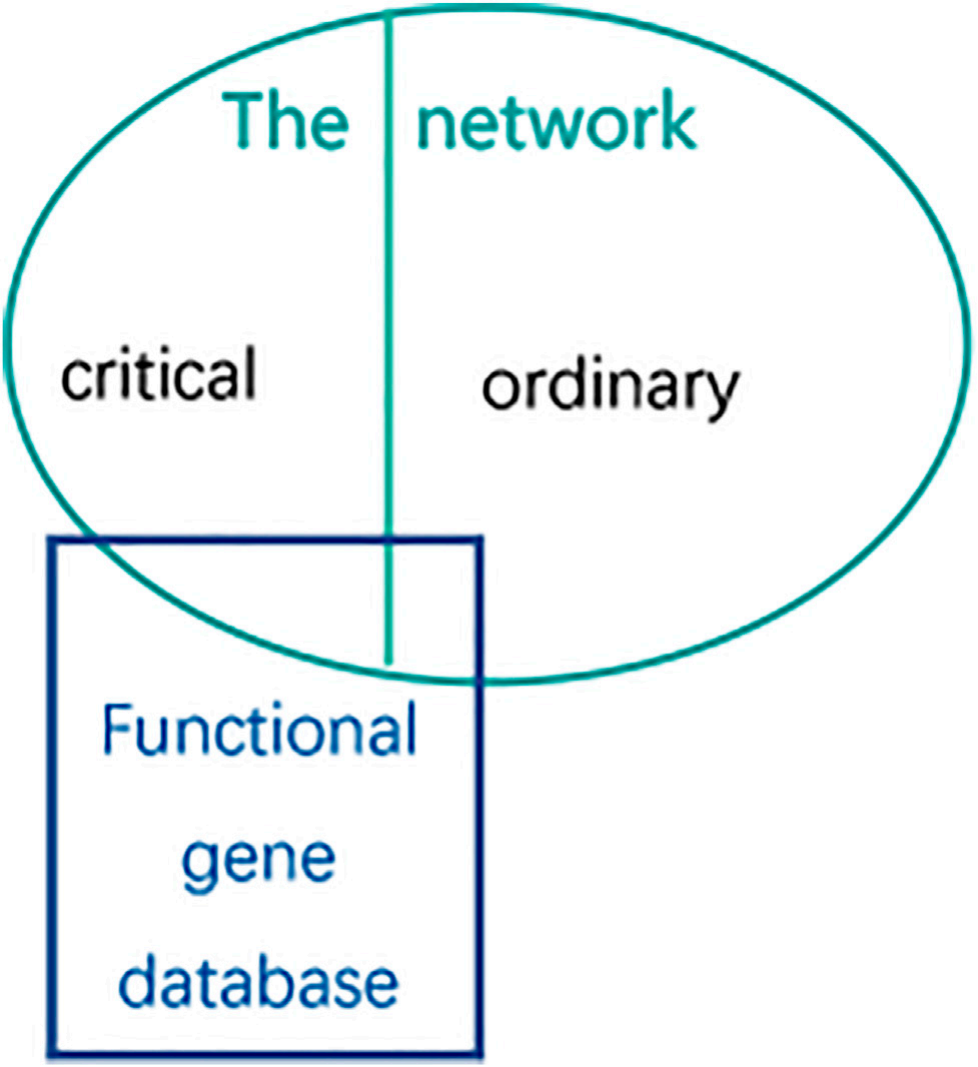
The method of Biological enrichment analysis. The circle represents genes of the network which represents forebrain GRN, hindbrain GRN and neuron associated cells cancer related GRN respectively. And the rectangle represents the functional gene database which is on behalf of essential genes, conserved genes, cancer genes or the related genes respectively.

As described in Figure 6, the oval part represents the gene set of the network under study, which is represented by *S*. here, it refers to the genes that need to be analyzed in the brain gene regulatory network (the network can be the forebrain GRN, hindbrain GRN and neuron associated cells cancer related GRN respectively; the gene can be the critical gene set or the ordinary gene set). The rectangular part represents some known functional gene databases, such as essential genes, cancer genes, conserved genes or other disease-related genes respectively, which is represented by *DB*. Overlap means that there will be some intersection of genetic data between them. Biological enrichment score calculating analysis is used to quantify the ratio of the critical genes after classification to the known functional gene databases.

Then the *z* score is calculated to estimate the enrichment by the Eq. 3:

$$\text{z}\;score=\frac{\left(S_{DB}-mean\;of\;R_{DB}\right)}{\mathrm{SD}\;of\;R_{DB}}$$ (3)

where *S*_*DB*_ is the number of genes in the intersection of set *DB* and *S*. *R*_*DB*_ is the number of intersection genes between the set *DB* and the extracted gene set, which are extracted *S*_*DB*_ genes randomly from *S*. And the mean of *R*_*DB*_ is the mean value of *R*_*DB*_ calculated after 1,000 random samples, while SD of *R*_*DB*_ is the standard deviation calculated after 1,000 random samples. It’s obvious that critical genes are rich in database *DB* if *z* score is bigger than 0.

Essential genes are obtained from DEG database, whose number is 8,254 for human (Zhang et al., 2004). Cancer genes are in COSMIC database (Futreal et al., 2004, and we collect 616 cancer genes for our work. Finally, the related disease genes are found in the Gene database (NCBI, https://www.ncbi.nlm.nih.gov/).

### 4.5 Survival Analysis

**FIGURE 7:**
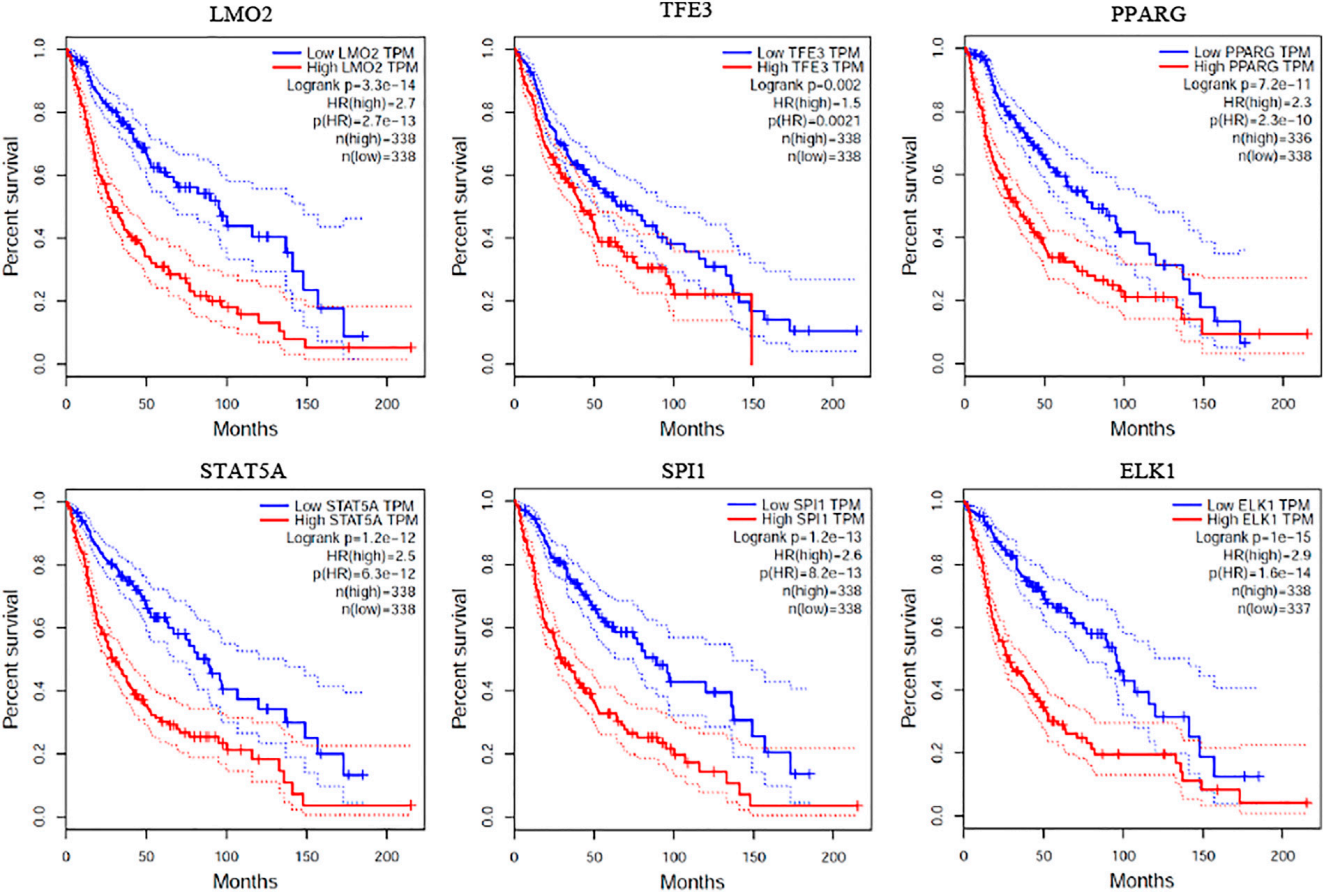
Survival analysis.

To validate whether the expression of critical genes are related to the survival time of prognosis, we conducted survival analysis by GEPIA (Tang et al., 2017). In the tool, we selected two brain-correlated cancers including brain lower grade glioma (LGG) and glioblastoma multiforme (GBM) from TCGA for analysis. The median value of the gene was selected as a cut-off to divide the samples into high and low expression group. As shown in Figure 7, the top 6 critical genes are highly related to the survival time of prognosis on both LGG and GBM. This indicates that the identified critical genes based on structural controllability analysis may also serve as potential biomarkers for the survival time of prognosis.

## 5 Conclusion

The comparison of neuron associated cells cancer related GRN and normal brain-specific GRNs (including forebrain and hindbrain GRN) shows that the neuron-related cell cancer-related gene regulatory network is more robust than other types. In order to obtain more network analysis results, we can consider combining disease-gene relationship data with gene-gene action relationship data to further improve the network topology and effective description, and explore more pathogenic mechanisms. At the same time, in terms of biological function analysis, we can further explore the biological function significance of the screened genes through more biological function analysis methods, so as to provide more specific tips for the research of glioma.

## Data Availability

The original contributions presented in the study are included in the article/Supplementary Material, further inquiries can be directed to the corresponding author.
