# Supplementary material for: Identification of Biomarker in Brain-specific Gene Regulatory Network Using Structural Controllability Analysis
Source: Front Bioinform. 2022 Jan 26;2:812314. doi: 10.3389/fbinf.2022.812314 (PMC9580899; doi:10.3389/fbinf.2022.812314)
Supplement: Supplementary file 1 [file DataSheet1.PDF]

Supplementary Material

1 SUPPLEMENTARY TABLES AND FIGURES

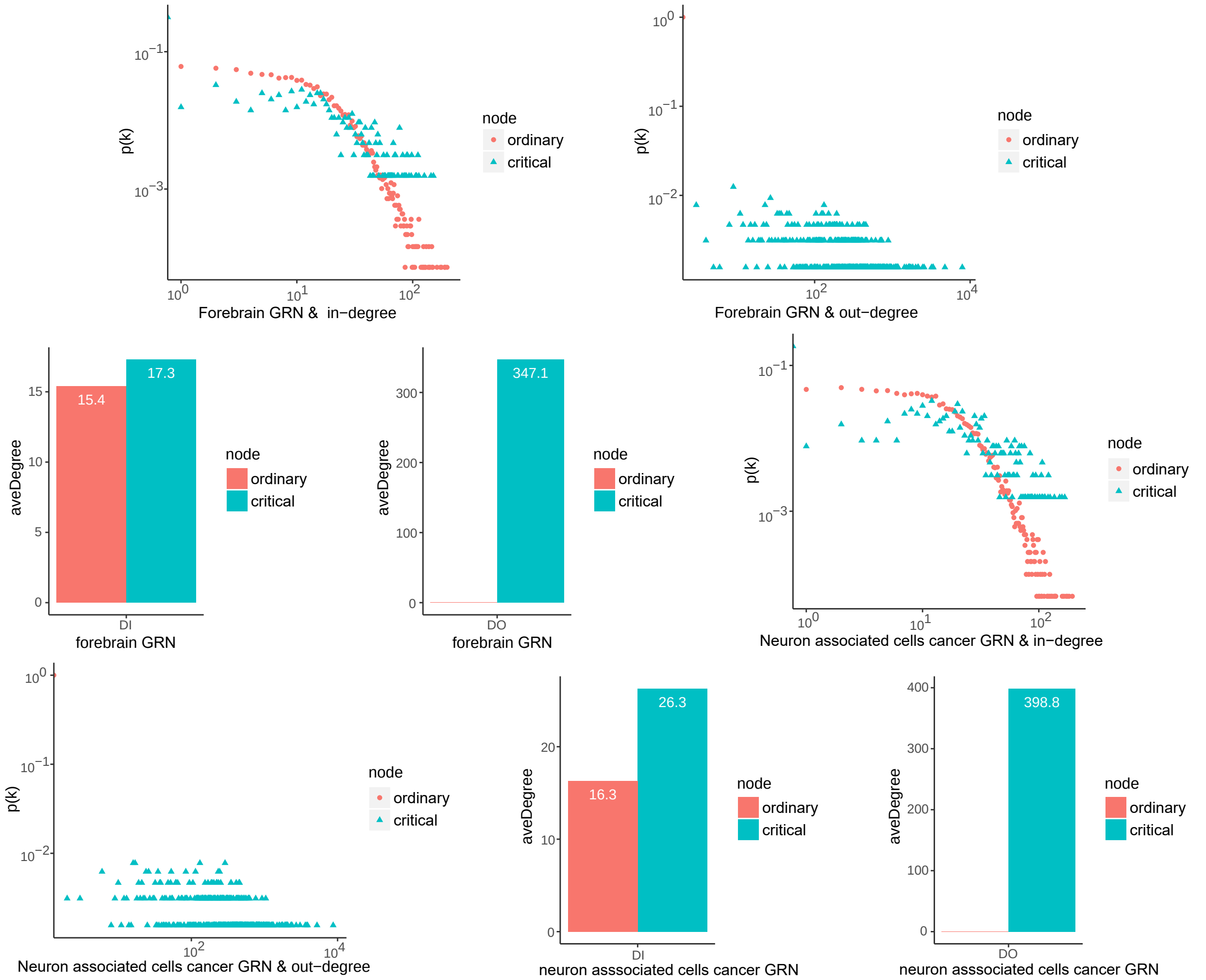

Figure S1. Characterizing the controllability of human brain-specific gene regulatory network and topological analysis. (a)In-degree distribution of forebrain GRN. (b)Out-degree distribution of forebrain GRN. (c)Average in-degree for ordinary and critical nodes in forebrain GRN. (d)Average out-degree for ordinary and critical nodes in forebrain GRN.(e)In-degree distribution of neuron associated cells cancer related GRN. (f)Out-degree distribution of neuron associated cells cancer related GRN. (g)Average in-degree for ordinary and critical nodes in neuron associated cells cancer related GRN. (h)Average out-degree for ordinary and critical nodes in neuron associated cells cancer related GRN.

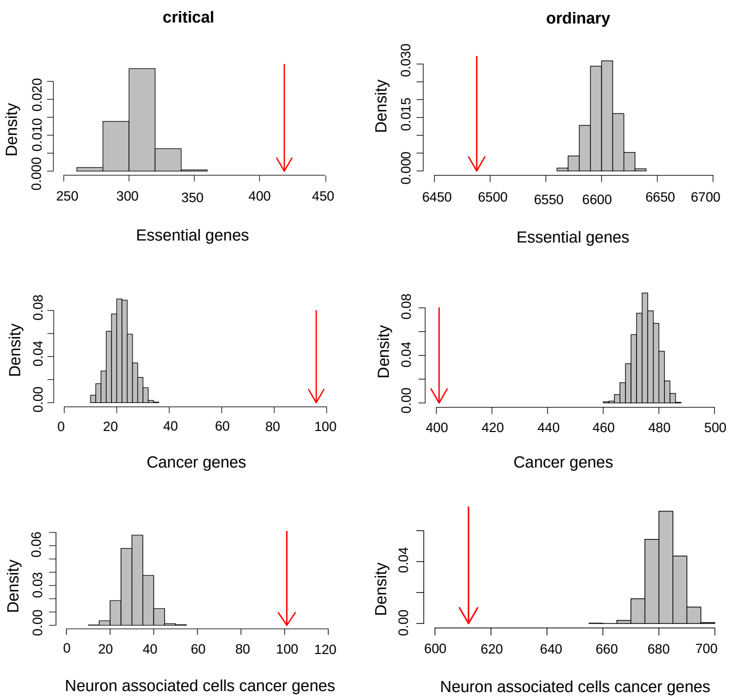

Figure S2. Biological enrichment of forebrain GRN. (a)Biological enrichment analysis of essential genes. Numbers of essential genes overlapping with critical and ordinary nodes are shown in red arrows. (b)Biological enrichment of cancer genes. Numbers of cancer genes overlapping with critical and ordinary nodes are shown in red arrows. (c)Biological enrichment analysis of neuron associated cells cancer genes. Numbers of these genes overlapping with critical and ordinary nodes are shown in red arrows.

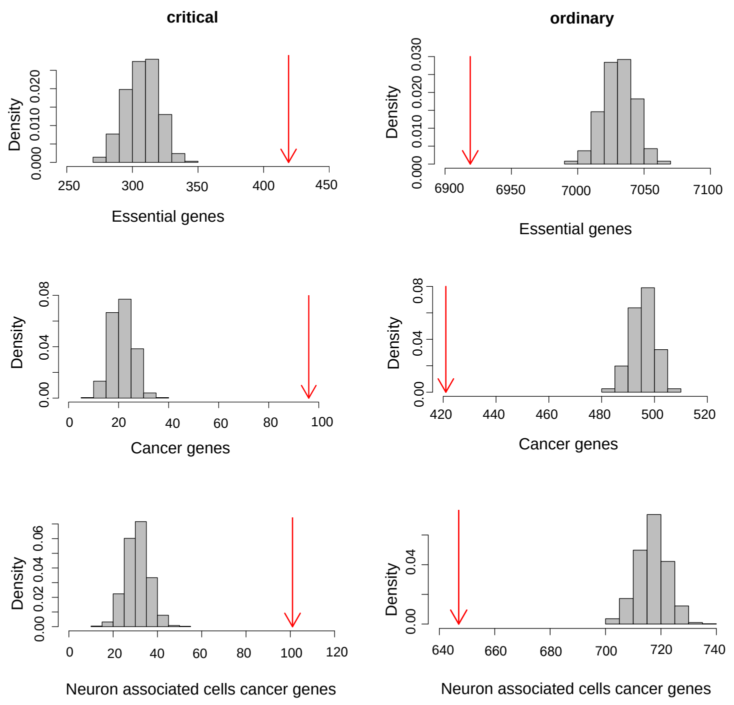

Figure S3. Biological enrichment analysis of neuron associated cells cancer related GRN. (a)Enrichment analysis of essential genes. Numbers of essential genes overlapping with critical and ordinary nodes are shown in red arrows. (b)Enrichment analysis of cancer genes. Numbers of cancer genes overlapping with critical and ordinary nodes are shown in red arrows. (c)Enrichment analysis of neuron associated cells cancer genes. Numbers of these genes overlapping with critical and ordinary nodes are shown in red arrows.

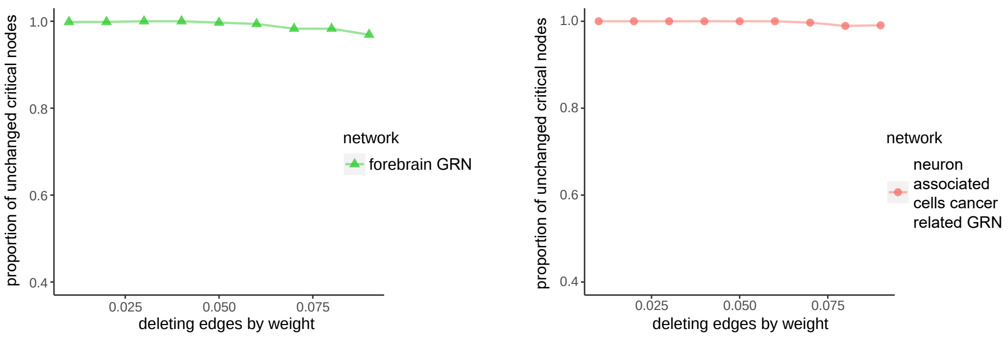

Figure S4. Robustness analysis of node classification. (a)Plot showing the fraction of critical nodes in new networks that overlaps with the original forebrain gene regulatory network, the new networks are obtained by deleting edges from original hindbrain gene regulatory networks. (b)Plot showing the fraction of critical nodes in new networks that overlaps with the original neuron associated cells cancer related gene regulatory network, the new networks are obtained by deleting edges from original hindbrain gene regulatory networks.

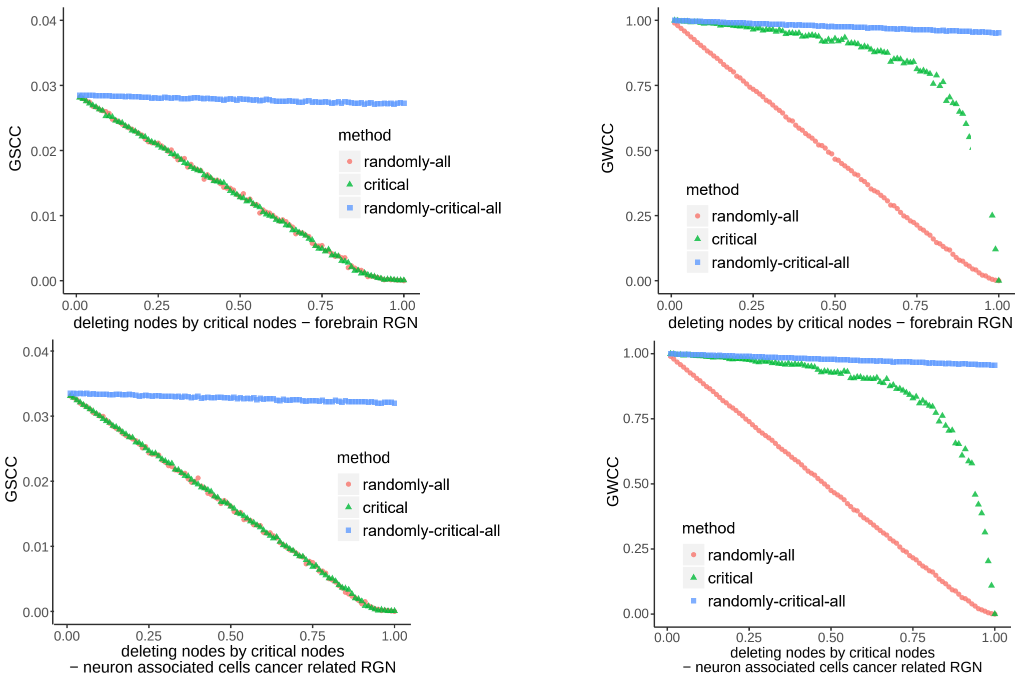

Figure S5. Robustness analysis of node classification. (a)The change of giant strongly connected component(GSCC) in forebrain GRN with three methods that are different in the way of deleting critical nodes. (b)The change of giant weakly connected component(GWCC) in forebrain GRN with three methods that are different in the way of deleting critical nodes. (c)The change of giant strongly connected component(GSCC) in neuron associated cells cancer related GRN with three methods that are different in the way of deleting critical nodes. (d)The change of giant weakly connected component(GWCC) in neuron associated cells cancer related GRN with three methods that are different in the way of deleting critical nodes.
